# The retrospective research of enteral nutrition with medium-chain triglyceride and total parenteral nutrition support of postoperative chylothorax in adults

**DOI:** 10.1177/2050312120938221

**Published:** 2020-07-01

**Authors:** Jie Zheng, Ying-Yi Chen, Chun-Ying Zhang, Wen-Qian Zhang, Zhi-Yong Rao

**Affiliations:** 1 Department of Clinical Nutrition, West China Hospital of Sichuan University, Chengdu, China; 2 Department of Laboratory Medicine, West China Hospital of Sichuan University, Chengdu, China

**Keywords:** Chylothorax, medium-chain triglyceride, total parenteral nutrition

## Abstract

**Background:**

Chylothorax is caused by thoracic lymphatic system injuries that leads to the lymph extravasating into the thoracic cavity. There are few reports comparing the therapeutic effects of enteral nutrition with medium-chain triglyceride and total parenteral nutrition, and the results are inconsistent. Our study aimed to research the optimum nutrition support method for chylothorax.

**Study design:**

We retrospectively reviewed 35 chylothorax patients after heart and chest surgery from 2014 to 2018, at West China Hospital of Sichuan University, among them there were 27 post-heart surgery patients. We analyzed the therapeutic effects and costs of enteral nutrition with medium-chain triglyceride (E group) and total parenteral nutrition (T group) for chylothorax.

**Results:**

The results were similar in patients with all surgeries and patients with only post heart surgery. The total cost during hospitalization in E group was higher than T group (*P* < 0.01), whereas the nutrition support cost was lower (*P* < 0.001). The length of hospital stay was longer in E group than T group (*P* > 0.05). Time from admission to surgery was shorter and from surgery to chylothorax diagnosis was longer in E group compared with T group. Time to resolution and removal of drainage was shorter in E group than T group but the differences were not significant.

**Conclusion:**

The therapeutic effects in enteral nutrition with medium-chain triglyceride and total parenteral nutrition had no obvious differences. Moreover, enteral nutrition with medium-chain triglyceride is safer and more economical. Therefore, we suggest that enteral nutrition with medium-chain triglyceride could be the first choice to treat postoperative chylothorax when the gastrointestinal tract function is allowed, and this result could be considered for postoperative chylous ascites.

## Introduction

Chylous fistula is caused by injuries to the thoracic or abdominal duct and its branches, or larger lymphatic vessels that the lymph extravasates into the third gap. The lymph accumulation in the chest is called chylothorax, which causes severe respiratory, nutritional and immune disorders. It results in the triglyceride level of thoracic effusion to be greater than 110 mg/dL. Chylothorax is a complication with low incidences after heart, esophagus, lung cancer and mediastinal surgery.

The main treatments for chylothorax are etiology and conservative treatment. Etiology treatment includes thoracic duct ligation, tumor chemotherapy, radiotherapy, and so on. Conservative treatment includes: (1) drainage of the pleural cavity; (2) maintaining stable internal environment; (3) nutrition support; (4) applying antibiotics to prevent thoracic infections and (5) somatostatin or its analogue octreotide can be used for postoperative chylothorax.

Nutrition support includes total parenteral nutrition (TPN), limiting dietary fat and replacing the fat with medium-chain triglyceride (MCT) in diet or enteral nutrition (EN) supplement. Reducing or inhibiting the long-chain fatty acid intake and absorption by gastrointestinal tract can decrease the production of chyle. Successful resolution of chylous fistula was described with TPN (complete oral intake cessation)^1^ and low-fat diet^2^ and MCT plan^3,4^ with variable success rates in different studies, but the effective comparison of different nutrition support methods is few and inconsistent. U Solmaz et al.^5^ reported the median time to complete clinical response of chylous ascites in a high-protein, low-fat with MCT diet group was shorter than in TPN group, but G Tulunay et al.^6^ reported the opposite results. W Pan et al.^7^ reported the curative efficacies of EN with MCT and TPN were similar for chylous ascites, but MCT diet was the worst, and the MCT plans was more economical. Based on the previous studies, we analyzed the therapeutic effects and cost differences between EN with MCT and TPN for chylothorax.

## Patients and methods

We retrospectively analyzed the patients diagnosed with chylothorax from 2014 to 2018 in West China Hospital of Sichuan University. Clinical information was collected including age, sex, underlying diseases, nutrition management days and costs, duration and volume of chest tube drainage and the length of hospital stay.

### Inclusion criteria

(1) Positive in a qualitative test of thoracic effusion after heart and chest surgery; (2) nutrition support at least 3 days and (3) TPN should provide above 60% of the daily energy requirement and oral nutrition supplement should provide above 400 kcal/d.

### Exclusion criteria

(1) Incomplete key information; (2) change of nutrition support method; (3) stopping nutrition support because of non-therapeutic reasons; (4) self-intake foods that were out of our protocol; (5) drainage volume was greater than 1500 or 1000 mL/day more than 5 days or 500 mL/day more than 14 days^4^ before diagnosed chylothorax and (6) patient’s condition became worse or resulted in death.

### Trial grouping and our clinical experienced nutrition support method

Once the chylothorax was confirmed, nutritional interventions were carried out. The daily energy and protein requirements for adults refer to American Society for Parenteral and Enteral Nutrition (ASPEN) and European Society for Parenteral and Enteral Nutrition (ESPEN) guidelines that recommend 25–35 kcal/kg d × ideal body weight (IBW) and the energy supply could reduce or increase for obese or malnourished patients, and protein recommendation was 1.2–2.0 g/kg IBW.

All patients were initially treated with nothing by mouth (NPO), a low fat diet and TPN before 2016. After that we gradually applied EN with MCT formula and MCT diet based on recent researches. Patients with high drainage output after surgery were initially treated with TPN, and changed to EN with MCT and/or a low-fat diet when drainage decreased. Mild cases were initially treated with MCT with EN. Nutritional management was done according to the physician’s discretion and different situations of the patients during the clinical treatment. To balance comparability of the data, we chose patients that were admitted before 2016 for the TPN group.

In our study, patients received four types of nutrition support modalities: (1) EN + MCT: MCT was the only lipid source, usually for the patients who were unconscious or oral intake was low; (2) MCT diet: low-fat foods cooked by MCT, usually for the patients who can eat normally; (3) EN + MCT + MCT diet: EN + MCT was the supplement for the patients who ordered the MCT diet but couldn’t meet their nutrition requirements and (4) TPN: inpatients before 2016.

## Statistical analysis

Statistical analysis was analyzed by SPSS software, version 17.0. The data normality test was conducted using Shapiro-Wilk test. Parametric data are expressed as $\left(\bar{X}\pm S\right)$. The study used the two-independent samples test and Fisher test. *P*-value less than 0.05 was considered statistically significant.

## Results

**Figure 1.**
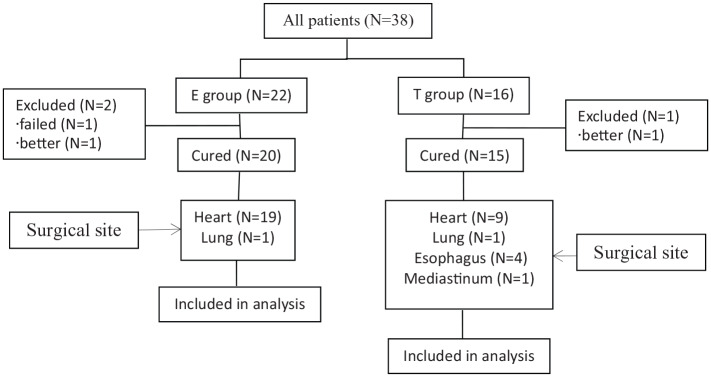
Study flow diagram.

Basic clinical characteristics and outcomes of two groups of 38 postoperative chylothorax patients were included in our study and 35 were cured, and there were no other complications in all patients. In E group, 21 patients underwent heart surgery, among them, one patient failed with conservative treatment, and one patient’s drainage volume declined after conservative treatment when she transferred to another department; one patient underwent lung surgery. In T group, nine patients underwent heart surgery, among them, one patient’s drainage volume declined when he discharged. The patients underwent four esophagus, one mediastinum and one lung surgery (Figure 1).

Among 35 cured patients included in the analysis, 15 patients took TPN and 20 patients took EN with MCT. Among 27 patients that underwent heart surgery, 8 patients took TPN and 19 patients took EN with MCT. There were no significant differences between the two groups in distribution of gender, age, body mass index (BMI) and thoracic drainage volume at the time diagnosed chylothorax both in patients who underwent all surgeries and the patients who only underwent heart surgery (Table 1).

**Table 1.** Basic clinical characteristics of two groups of patients.

		Gender (M/F)	Age (years)	BMI (kg/m^2^)	First day drainage volume (mL)
Patients with all surgeries	EN + MCT(*N* = 20)	11/9	52.80 ± 16.06	20.61 ± 3.12	373.53 ± 193.79 (*N* = 17)
	TPN(*N* = 15)	10/5	49.27 ± 14.88	20.99 ± 3.20	410.08 ± 193.40 (*N* = 12)
	*P* value	0.738	0.511	0.726	0.621
Patients post heart surgery	EN + MCT (*N* = 19)	10/9	52.26 ± 16.31	20.64 ± 3.20	371.88 ± 200.02 (*N* = 16)
	TPN (*N* = 8)	4/4	44.25 ± 12.74	20.03 ± 3.61	455.00 ± 184.24 (*N* = 5)
	*P* value	1.000	0.228	0.665	0.420

BMI: body mass index; EN: enteral nutrition; MCT: medium-chain triglyceride; TPN: total parenteral nutrition.

### Comparisons of clinical outcomes in patients with all surgeries cured with different nutrition support methods

**Figure 2.**
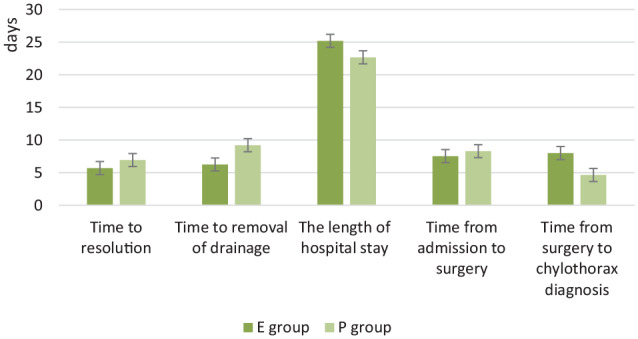
Clinical outcomes in patients with all surgeries.

There were 35 cured patients included in the analysis. The total hospitalization cost in E group was significantly higher than T group but the nutrition support cost was lower (*P* = 0.000). The length of hospital stay was longer in E group than T group (*P* > 0.05). The time from admission to surgery was shorter (*P* > 0.05) and from surgery to first sign of chylothorax was longer (*P* = 0.011) in E group compared with T group. The time to resolution and removal of drainage was shorter in E group than T group but the differences were not significant. There was no significant difference between the two groups of thoracic drainage volume during the first day after starting nutrition support (Table 2 and Figure 2).

**Table 2.** Clinical outcomes in patients with all surgeries in two groups (*N* = 35).

	EN + MCT (*N* = 20)	TPN (*N* = 15)	*P* value
Nutrition support cost (RMB)	459.35 ± 250.10	1808.68 ± 964.63	0.000
Total hospitalization cost (RMB)	180,321.32 ± 104,951.28	74,494.99 ± 43,332.40	0.001
Time to resolution (d)	5.70 ± 2.36	6.93 ± 3.97	0.260
Time to removal of drainage (d)	6.25 ± 2.10	9.20 ± 5.63	0.071
The length of hospital stay (d)	25.20 ± 10.70	22.67 ± 9.02	0.464
Time from admission to surgery(d)	7.53 ± 5.00	8.29 ± 4.82	0.654
Time from surgery to chylothorax diagnosis (d)	8.00 ± 3.93	4.64 ± 3.20	0.011
Second day drainage volume (mL)	220.31 ± 149.77 (*N* = 16)	222.00 ± 179.25 (*N* = 11)	0.979

EN: enteral nutrition; MCT: medium-chain triglyceride; TPN: total parenteral nutrition.

### Comparisons of clinical outcomes in post-heart surgery patients cured with different nutrition support methods

**Figure 3.**
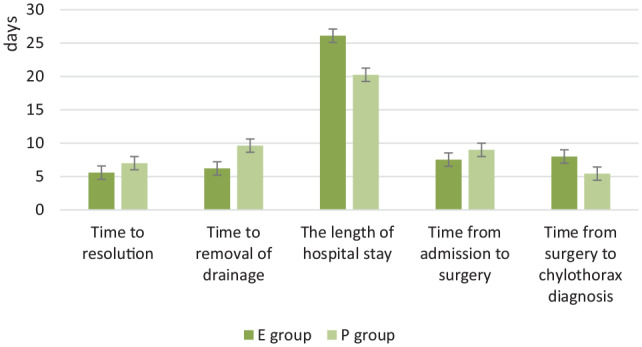
Clinical outcomes in patients post heart surgery.

Considering the differences in disease diagnoses might affect the homogeneity, we reanalyzed the 28 patients who only underwent heart surgery. Among the patients, 8 patients took TPN and 19 patients took EN with MCT. The total hospitalization cost in E group was significantly higher than T group but the nutrition support cost was lower (*P* *=* 0.000). The length of the hospital stay was longer in E group than T group (*P* > 0.05). The time from admission to surgery was shorter and from surgery to first sign of chylothorax was longer in E group compared with T group (*P* > 0.05). The time to resolution and removal of drainage was shorter in E group than T group but the differences were not significant (Table 3 and Figure 3).

**Table 3.** Clinical outcomes in patients post heart surgery in two groups (*N* = 28).

	EN + MCT (*N* = 19)	TPN (*N* = 9)	*P* value
Nutrition support cost (RMB)	449.11 ± 252.60	1490.25 ± 656.27	0.000
Total hospitalization cost (RMB)	189,428.44 ± 99,376.83	73,686.14 ± 36,853.62	0.004
Time to resolution (d)	5.58 ± 2.36	7.00 ± 4.93	0.315
Time to removal of drainage (d)	6.21 ± 2.15	9.63 ± 6.80	0.056
The length of hospital stay (d)	26.11 ± 10.18	20.25 ± 6.67	0.149
Time from admission to surgery(d)	7.53 ± 5.14	9.00 ± 5.66	0.514
Time from surgery to chylothorax diagnosis (d)	8.00 ± 4.04	5.43 ± 3.92	0.141

EN: enteral nutrition; MCT: medium-chain triglyceride; TPN: total parenteral nutrition.

## Comments

A few studies have compared the therapeutic effects of EN with TPN for chylous fistula. G Tulunay et al.^6^ showed that the median time to complete the clinical recovery was 28 days (range, 11–60) with a low-fat diet supplied with MCT and 10 days (range, 5–66) with TPN. U Solmaz et al.^5^ showed the median time to resolution was 3 days (range, 2–5 days) in diet group and 5.5 days (range, 3–12 days) in TPN group with significant difference. Our research showed the similar curative effect with U Solmaz et al. that the time to resolution in E group was statistically shorter than T group. There was no significant difference between EN with MCT group and TPN group in thoracic drainage volume at the time of chylothorax diagnosis and on the first day after starting nutrition support. But the thoracic drainage volume both decreased in all patients on the first day after starting nutrition support, which was different from the results of W Pan et al.

Nutritional optimization is one of the cornerstones of therapy for chylous fistula^8,9^. And emerging researches suggested that EN especially with MCT plan should be tried before TPN.^4,10–13^ On one hand, TPN may be associated with complications^13^ such as venous infection and abnormal liver function. In contrast, diet and EN have been reported to be safe and more natural. On the other hand, the cost of TPN is generally more expensive compared to EN. NA van der Gaag et al.^14^ supported this view. W Pan et al.^7^ reported the nutrition support cost of EN is lower than TPN. And our results were consistent with these previous reports.

In our study, the length of hospital stay in E group was longer than T group. We found the time from admission to surgery in E group was similar with T group but from surgery to chylothorax diagnosis in E group was longer than T group. NA van der Gaag et al.^14^ reported that chylous ascites was diagnosed on postoperative day 6 (range, 5–8). The time from surgery to chylothorax diagnosis in our study was 8.00 ± 3.93 days (E group) and 4.64 ± 3.20 days (T group). This might partly explain the total cost during hospitalization was higher in E group. Isolated chylous ascites was associated with prolonged hospital stays, hence it showed us that diagnosing chylothorax earlier might contribute to decrease the length of hospital stay and the total hospitalization cost.

The results in our study were similar in patients with all surgeries and the patients with only post heart surgery. This further proved the validity of our clinical protocol for the nutrition support of chylothorax and our clinical protocol may also be useful for chylous fistula caused by other surgical sites.

Limitations of this study include: The samples were from a single institution. We collected all information of the patients diagnosed with chylothorax who met the inclusion criteria in our hospital from 2014 to 2018, but the sample size wasn’t calculated before the research. This study was a retrospective study. Relevant information, such as the daily chest drainage output was only partially recorded. We did not analyze the patients treated with a combination of EN and PN because of the cases were few. The diagnosis of chylothorax used a qualitative test which might result in lower sensitivity.

## Conclusion

In conclusion, first, the therapeutic effects had no obvious differences. Second, oral intake and EN has fewer associated complications. Finally, the nutrition support cost would be significantly decreased. Therefore, our research suggests that EN with MCT should be the first choice to treat postoperative chylothorax when the gastrointestinal tract function is allowed and the chest drainage volume is below 1500 mL/day, and this result could be considered for postoperative chylous ascites.
